# Routine Immediate Lung Assessment During CT Conceived for Other Purposes (Thoracic Spine CT, Simulation CT for Radiotherapy, PET-CT): A Costless Screening and Surveillance Tool for Lung Opacities in the COVID-19 Era

**DOI:** 10.1017/dmp.2020.427

**Published:** 2021-08

**Authors:** Antonio Manca, Saverio Bellizzi, Marco Gatti, Manuela Racca, Delia Campanella, Gabriele Chiara, Daniele Regge

**Affiliations:** 1 Candiolo Cancer Institute, Radiology Unit, FPO, IRCCS, Candiolo, Italy; 2 Medical Epidemiology, Independent Consultant, Geneva, Switzerland; 3 Candiolo Cancer Institute, Radiation Oncology Unit, FPO, IRCCS, Candiolo, Italy; 4 Candiolo Cancer Institute, Nuclear Medicine Unit, FPO, IRCCS, Candiolo, Italy

**Keywords:** health care facilities, manpower, services, infection control, public health

Early computed tomography (CT) changes have been reported in asymptomatic (subclinical) patients, as well as in patients with initial false-negative reverse transcription polymerase chain reaction (RT-PCR) results.^1^ Thus, despite the use of CT as a screening tool not being advised in European guidelines,^2^ active surveillance via CT scan routinely performed for other purposes might be reasonable.

Candiolo Cancer Institute is an oncologic research and treatment hospital that has been designated as a “COVID free” facility amid the regional health system reconfiguration to cope with the epidemic. As such, the institute is currently hosting the uninfected oncologic patients of other nearby re-purposed health facilities of the Piemonte region that is currently the second Italian region for confirmed cases (almost 30 000).

In order to reduce the possibility of infection diffusion, severe acute respiratory syndrome coronavirus 2 (SARS-CoV-2) swab testing is routinely performed in every inpatient before hospitalization. In every outpatient undergoing a routine chest CT, even if included in staging imaging, lung parenchyma is immediately evaluated before the patient leaves the room to rule out a suspicious coronavirus disease (COVID-19) case; the radiologist also calls the referring clinician and the hospital health management in order to handle the case with prophylactic isolation of the patient in a dedicated internal facility and swab testing. In case clinical, lab, and radiological features suggest a SARS-CoV-2 infection, the patient can be transferred to a COVID-19 facility. The number of early diagnosis and isolation of potential COVID-19 cases might be further increased if the assessment of the lung is included in imaging conceived for other finalities; our purpose is to turn this “missed opportunity” into a screening tool with no added costs and radiation exposure. Specifically, we suggest an optimization of the regular use of CT scan of the thoracic spine (but also rib cage and sternum), CT simulation for radiotherapy of spine, breast, and other thoracic structures, and a positron emission tomography (PET-CT) to obtain an early detection and assessment of suspicious opacities in the lungs.

The CT scan required for the study of the thoracic spine is more commonly performed with a narrow field of view (FOV) during free breathing and the evaluation of the bone is commonly made with “bone window”; at our institution, due to the wide experience in percutaneous vertebroplasty (PV), we already perform full FOV scans of the thorax during breath hold in order to evaluate also lungs and any extraspinal incidental findings as part of preoperative imaging (when a spine MRI is not feasible or not sufficient). Since the onset of the COVID-19 outbreak, this consolidated practice has become even more useful considering the need for swab testing for patients set to be hospitalized for PV. Thoracic simulation CT performed for radiotherapy is usually performed during superficial free-breathing and most times is not visualized with “lung window.” The usefulness of this evaluation has already been reported,^3^ and, even in our institution, the first patient diagnosed for COVID-19 was detected with a simulation CT. Lung parenchyma can be evaluated also with PET-CT and, even with some limitations due to motion artifacts, potential COVID-19 patients can be identified within the framework of tumoral staging and follow-up.^4^

In our opinion, screening patients already scheduled for a CT by expanding the diagnostic target to the lung parenchyma can provide an opportunity to optimize the prevention and control strategy for the considerable portion of patients showing every day. Moreover, the early detection and isolation of potential COVID-19 patients allow the proper and prompt decontamination of rooms and equipment that in radiology have a high turnover of patients while in nuclear medicine and radiotherapy imply prolonged and/or repeated occupation of the rooms. Radiologists can play a key role in promoting enhanced screening use of CT, by both engaging medical-technical staff of Radiology, Nuclear Medicine, and RT Departments, as well as sharing their expertise to evaluate suspicious cases.

The absence of additional costs and radiation exposure makes this practice promptly feasible without ethical and financial implications and provides an immediate opportunity to detect, isolate, and manage infected patients, thus breaking the transmission chain.
